# The group II metabotropic glutamate receptor agonist LY354740 and the D2 receptor antagonist haloperidol reduce locomotor hyperactivity but fail to rescue spatial working memory in GluA1 knockout mice

**DOI:** 10.1111/ejn.13539

**Published:** 2017-03-04

**Authors:** Thomas Boerner, Alexei M. Bygrave, Jingkai Chen, Anushka Fernando, Stephanie Jackson, Chris Barkus, Rolf Sprengel, Peter H. Seeburg, Paul J. Harrison, Gary Gilmour, David M. Bannerman, David J. Sanderson

**Affiliations:** ^1^ Department of Experimental PsychologyUniversity of Oxford9 South ParksOxfordOX1 3UDUK; ^2^ Max Planck Research GroupInstitute for Anatomy and Cell Biology, Heidelberg UniversityHeidelbergGermany; ^3^ Department of PsychiatryUniversity of OxfordWarneford HospitalOxfordUK; ^4^ Lilly Centre for Cognitive NeuroscienceDiscovery BiologyLilly Research CentreLilly UKWindleshamSurreyUK; ^5^ Department of PsychologyDurham UniversityScience Site, South RoadDurhamDH1 3LEUK

**Keywords:** AMPA, Gria1, habituation, schizophrenia

## Abstract

Group II metabotropic glutamate receptor agonists have been suggested as potential anti‐psychotics, at least in part, based on the observation that the agonist LY354740 appeared to rescue the cognitive deficits caused by non‐competitive *N*‐methyl‐d‐aspartate receptor (NMDAR) antagonists, including spatial working memory deficits in rodents. Here, we tested the ability of LY354740 to rescue spatial working memory performance in mice that lack the GluA1 subunit of the AMPA glutamate receptor, encoded by Gria1, a gene recently implicated in schizophrenia by genome‐wide association studies. We found that LY354740 failed to rescue the spatial working memory deficit in *Gria1* ^*−/−*^ mice during rewarded alternation performance in the T‐maze. In contrast, LY354740 did reduce the locomotor hyperactivity in these animals to a level that was similar to controls. A similar pattern was found with the dopamine receptor antagonist haloperidol, with no amelioration of the spatial working memory deficit in *Gria1* ^*−/−*^ mice, even though the same dose of haloperidol reduced their locomotor hyperactivity. These results with LY354740 contrast with the rescue of spatial working memory in models of glutamatergic hypofunction using non‐competitive NMDAR antagonists. Future studies should determine whether group II mGluR agonists can rescue spatial working memory deficits with other NMDAR manipulations, including genetic models and other pharmacological manipulations of NMDAR function.

## Introduction

Group II metabotropic glutamate receptor agonists have been suggested as potential anti‐psychotics, and have been used in clinical trials, albeit with mixed results (Patil *et al*., 2007; Kinon *et al*., 2015). Interest in these compounds as anti‐psychotics was initially raised by the observation that group II mGluR agonists, such as LY354740, were found to rescue the deficit in spatial working memory seen following glutamatergic dysfunction with non‐competitive NMDAR antagonists, like phencyclidine (PCP) and MK‐801, on the T‐maze rewarded alternation task in rats (Moghaddam & Adams, 1998; Blot *et al*., 2015), as well as reducing the locomotor hyperactivity seen with both dopaminergic and glutamatergic challenges (Moghaddam & Adams, 1998; Pehrson & Moghaddam, 2010). These compounds were therefore attractive as they appeared to provide a way of restoring cognitive function in disorders like schizophrenia, as well as ameliorating positive symptoms (Sendt *et al*., 2012).

The underlying rationale for these studies was provided by the influential glutamate hypothesis of schizophrenia, in which NMDAR dysfunction plays a central role (Olney & Farber, 1995; Coyle, 1996), and has been supported by recent evidence that schizophrenia genes impact on glutamatergic synapses (Harrison & Owen, 2003; Moghaddam, 2003; Harrison & Weinberger, 2005; Harrison & West, 2006). Despite the focus on NMDARs, there is increasing evidence that AMPARs, and in particular the GluA1 subunit (also known as GluR‐A or GluR1, and encoded by the gene *Gria1*), may also contribute to the disorder (Barkus *et al*., 2014). Post‐mortem studies have suggested that GluA1 expression is decreased in the hippocampus of patients with schizophrenia (e.g. Eastwood *et al*., 1995) and, significantly, recent large scale GWAS meta‐analyses have established genome‐wide association to schizophrenia for the *Gria1* locus (Ripke *et al*., 2013; Schizophrenia Working Group of the Psychiatric Genomics, 2014).

Mice in which the *Gria1* gene has been deleted (*Gria1* ^*−/−*^ mice) show impaired hippocampal synaptic plasticity (Zamanillo *et al*., 1999; Hoffman *et al*., 2002; Romberg *et al*., 2009; Erickson *et al*., 2010). At the behavioural level, *Gria1* ^*−/−*^ mice exhibit a number of robust and reproducible phenotypes, including pronounced spontaneous locomotor hyperactivity in novel environments, although activity levels are not elevated in the home cage (Bannerman *et al*., 2004; Wiedholz *et al*., 2008; Procaccini *et al*., 2013; Maksimovic *et al*., 2014). *Gria1* ^*−/−*^ mice also exhibit a selective short‐term memory deficit on hippocampus‐dependent spatial working memory, win‐shift maze tasks, including T‐maze rewarded alternation (Reisel *et al*., 2002; Schmitt *et al*., 2003; Sanderson *et al*., 2007; Taylor *et al*., 2011). We have shown previously that *Gria1* ^*−/−*^ mice exhibit impaired performance on this task, even after extensive training (Reisel *et al*., 2002). In contrast, *Gria1* ^*−/−*^ mice display normal (Zamanillo *et al*., 1999; Reisel *et al*., 2002) or even, enhanced (Schmitt *et al*., 2003; Sanderson *et al*., 2009) long‐term spatial memory performance. We have proposed that the selective deficit in tests of spatial working memory reflects a failure to express short‐term habituation to recently experienced stimuli (Sanderson & Bannerman, 2012). This account is also able to explain other behavioural effects found with GluA1 knockout using nonspatial stimuli (Sanderson *et al*., 2011a,b).

The group II mGluR agonist LY354740 has been shown to reduce the locomotor hyperactivity in *Gria1* ^*−/−*^ mice (Procaccini *et al*., 2013). Therefore, in the present study we investigated if LY354740 would rescue the spatial working memory deficit in *Gria1* ^*−/−*^ mice, given its apparent pro‐cognitive effects in other models of glutamatergic hypofunction (Moghaddam & Adams, 1998; Blot *et al*., 2015), and its effects on locomotor activity in *Gria1* ^*−/−*^ mice (Procaccini *et al*., 2013). Furthermore, Wiedholz *et al*. (2008) demonstrated that a 0.3 mg/kg dose of haloperidol (Mohn *et al*., 1999), a dopamine receptor antagonist and widely used antipsychotic drug, also reduces the locomotor hyperactivity exhibited by *Gria1* ^*−/−*^ mice. Therefore, for comparison, we also investigated whether this same dose of haloperidol that reduces the locomotor hyperactivity seen in *Gria1* ^*−/−*^ mice would rescue spatial short‐term/working memory performance in these mice on a T‐maze rewarded alternation task.

## Methods

### Subjects

The experiments used littermate, aged‐matched wild‐type (WT) and *Gria1* ^*−/−*^ mice bred in the Department of Experimental Psychology at the University of Oxford (see Zamanillo *et al*. (1999) for details of genetic construction, breeding and subsequent genotyping). In brief, the mice were derived from 129S2/SvHsd and C57BL/6JolaHsd background strains, and then backcrossed to C57BL/6JolaHsd. Experimental cohorts were generated by crossing male and female heterozygous *Gria1* ^+/−^ mice. The mice were 6–14 months old at the start of testing. Mice were housed in cage groups of up to 6, in a temperature and humidity controlled vivarium. The animals were maintained on a 12 h light‐dark cycle (lights on; 07.00 – 19.00). All procedures were in accordance with the United Kingdom Animals Scientific Procedures Act (1986); under the UK Home Office project licenses PPL 30/2561 and PPL 30/3068.

### Drugs

In Experiments 1 and 2, LY354740 (provided by Eli Lilly U.K.) was made up in sterile water (Norbrook) at a concentration of either 1.5 or 3 mg/mL. The pH was adjusted to approximately pH 7 by spiking with small aliquots of 1M NaOH. All injections were given intraperitoneally at doses of either 15 or 30 mg/kg/10 mL. These doses have been used in prior studies examining the effects of group II mGluR agonists on locomotor hyperactivity in *Gria1* ^*−/−*^ mice (see Procaccini *et al*., 2013). In Experiments 3 and 4, haloperidol (0.3 mg/kg/10 mlL/; Janssen‐Cilag Ltd, High Wycombe, UK) or saline injections were given intraperitoneally. The drug dose was selected on the basis of the previous study by Wiedholz *et al*. (2008), in which haloperidol was shown to reduce hyperactivity in *Gria1* ^*−/−*^ mice.

### Experiment 1: The effect of the group II mGluR agonist LY354740 on spatial short‐term/working memory during rewarded alternation testing in Gria1^*−/−*^ mice

We first assessed the effects of LY354740 on spatial working memory performance during rewarded alternation testing in wild‐type and *Gria1* ^*−/−*^ mice. Rewarded alternation (see Reisel *et al*., 2002; Bannerman *et al*., 2003; Schmitt *et al*., 2004; Deacon & Rawlins, 2006; Taylor *et al*., 2011) was tested using a grey, elevated wooden T‐maze. This consisted of a start arm (47 × 10 cm) and two identical goal arms (35 × 10 cm), surrounded by a 10 cm high wall. A metal food well (0.8 cm diameter, 0.6 cm high) was fixed to the end of each goal arm. Several days before the start of testing, mice were reduced to 85% of their free‐feeding weight, by receiving a restricted diet, and familiarized to the T‐maze, and to the sweetened, condensed milk reward (condensed milk diluted with water using a 50 : 50 ratio).

Each trial consisted of a sample run and a choice run. On the sample run a mouse was placed in the start arm, facing away from the goal arms, and forced to enter either the left goal arm or right goal arm (determined by blocking the entrance to the opposite goal arm with a wooden block) to obtain a milk reward (0.1 mL). After the sample run the block was removed and the mouse was placed back in the start arm and allowed to choose to enter either the left or right goal arm (free choice run). The mouse was rewarded for choosing the previously unvisited arm (i.e., for alternating). A choice was made once all four paws were inside a goal arm. There was a minimal delay between the end of the sample run and start of the choice run (< 5 s). This reflected the minimum amount of time required for the experimenter to remove the animal from the maze, remove the wooden block and then replace the mouse at the beginning of the start arm. On sample runs mice were forced either left or right according to a pseudorandom sequence in which there were equal numbers of left and right forced runs per blocks of ten trials, and no more than three consecutive sample runs to the same goal arm. The inter‐trial interval (i.e., the interval between the end of a choice run and the next, subsequent sample run) was approximately 6–10 min.

In Experiment 1A wild‐type (female: *N* = 6; male: *N* = 5) and *Gria1* ^*−/−*^ mice (female: *N* = 7; male: *N* = 8; see Table 1) first received 30 trials of drug‐free testing (five trials per day for 6 days). Each animal was then tested on rewarded alternation following an injection of either LY354740 (15 mg/kg) or vehicle. After injection animals were returned to the home cage for 30 min before behavioural testing commenced. Each animal was given 10 trials of rewarded alternation in the T‐maze. Mice were given a maximum of 120 s to a complete a trial. At least 24 h after the first round of drug testing the animals were re‐tested in the absence of any drug treatment to ensure that there were no long‐term effects of the drug, and that the mice maintained a high level of alternation performance (with three sessions of five trials per day in Experiment 1A, and one session of 10 trials per day in Experiments 1B and 1C). Twenty‐four hours after this re‐testing, mice received a further 10 trials of rewarded alternation testing, but now under the drug condition that they had not previously received (i.e., mice that had previously received LY354740 then received vehicle injections, and vice versa for the remaining mice). The order of drug exposure was counterbalanced within genotype and sex as far as possible given the numbers of mice. In all stages, the number of trials in which the animal alternated, as well as time taken to run from the start arm to the food well on the sample run (sample latency), and the time taken to run from the start arm to making a choice (i.e., entering an arm with all four paws) on the choice run (choice latency), were recorded. Latencies were measured by the experimenter using a stopwatch. The experimenter was blind to the genotype and drug allocations of the animals throughout testing.

**Table 1:** Details of experimental procedure, age and sex of mice, drug manipulation and dose, and feeding regime for Experiments 1–4

	Procedure	Age	Sex	Drug manipulation	Feeding regime
Experiment 1A	Rewarded alternation –long inter‐trial interval	12–14 months	WT: female = 6, male = 5 Gria1^*−/−*^: female = 7, male = 8	LY35470 15 mg/kg within‐subjects	Food restriction
Experiment 1B	Rewarded alternation –long inter‐trial interval	6–10 months	WT: male = 7 Gria1^*−/−*^: male = 7	LY35470 30 mg/kg within‐subjects	Food restriction
Experiment 1C	Rewarded alternation –short inter‐trial interval	Same mice as Experiment 1B	Same mice as Experiment 1B	LY35470 30 mg/kg within‐subjects	Food restriction
Experiment 2A	Locomotor activity	Same mice as Experiment 1A	Same mice as Experiment 1A	LY35470 15 mg/kg within‐subjects	Free‐feeding
Experiment 2B	Locomotor activity	9–12 months	WT: female = 4, male = 6 Gria1^*−/−*^: female = 6, male = 5	LY35470 30 mg/kg within‐subjects	Free‐feeding
Experiment 2C	Locomotor activity	Same mice as Experiment 1B and 1C	Same mice as Experiment 1B and 1C	LY35470 30 mg/kg within‐subjects	Food restriction
Experiment 3	Rewarded alternation –long inter‐trial interval	9 months	WT: female 6, male = 6 Gria1^*−/−*^: female = 6, male = 6	Haloperidol 0.3 mg/kg within subjects	Food restriction
Experiment 4	Locomotor activity	Same mice as Experiment 3	Same mice as Experiment 3	Haloperidol 0.3 mg/kg between‐subjects (genotype and sex was balanced across drug conditions)	Free‐feeding

In Experiment 1B, separate groups of male wild‐type (*N* = 7) and *Gria1* ^*−/−*^ mice (*N* = 7) underwent the same procedure as Experiment 1A but now they received either vehicle or a higher dose of LY354740 (30 mg/kg; see Table 1). Subsequently, in Experiment 1C, to investigate the potential effects of increased proactive interference, the procedure used in Experiment 1B was repeated in the same mice, using the same drug dose (30 mg/kg), but now using a modified testing protocol in which the interval between trials (i.e. the interval between the end of a choice run and the next, subsequent sample run) was reduced to 20 s (see Table 1). This procedure more closely matches the behavioural paradigm used by Moghaddam & Adams (1998) in which LY354740 reversed the effects of the non‐competitive NMDAR antagonist, phencyclidine.

### Experiment 2: The effect of the group II mGluR agonist LY354740 on spontaneous locomotor activity in Gria1^*−/−*^ mice

We next assessed the effects of LY354740 on spontaneous locomotor activity in a novel environment in wild type and *Gria1* ^*−/−*^ mice. In Experiment 2A the mice that had previously been tested in Experiment 1A were returned to a free feeding regime and then tested for spontaneous locomotor activity (see Table 1) in clear plastic cages (26 × 16 × 17 cm), containing clean sawdust (see Bannerman *et al*., 2004). Activity levels were assessed by an array of infrared photobeam sensors that covered the x and y axes of the cages (San Diego Instruments). Immediately after an injection of either 15 mg/kg LY354740 or vehicle mice were placed individually into test cages. The beam breaks were recorded in eight bins of 15 min over a 2‐h session. After the first round of locomotor testing (and following a washout period of 7 days), mice were tested in the locomotor cages with the vehicle/drug allocations reversed. In the second test mice were allocated a cage that was different from the first test. The order of drug/vehicle injections was counterbalanced for sex and genotype as far as possible given the number of mice per condition.

In Experiment 2B experimentally naïve wild‐type (female: *N* = 6; male: *N* = 6) and *Gria1* ^*−/−*^ mice (female: *N* = 6; male: *N* = 5) that had been maintained with *ad libitum* access to food were also tested for locomotor activity using an identical protocol as in Experiment 2A, but now with vehicle and 30 mg/kg LY354740 (see Table 1).

Finally, in Experiment 2C the mice that had been previously used in Experiments 1B and 1C were maintained on food restriction (to match the conditions used for T‐maze testing) and locomotor activity was assessed with either the 30 mg/kg dose of the drug or vehicle as described above (see Table 1).

### Experiment 3: The effect of haloperidol on spatial short‐term/working memory during rewarded alternation testing in Gria1^*−/−*^ mice

For comparison, we also investigated the effects of the anti‐psychotic, D2 receptor antagonist haloperidol on spatial working memory performance in wild‐type and knockout mice (see Table 1). Wild‐type (female: *N* = 6; male: *N* = 6) and *Gria1* ^*−/−*^ mice (female: *N* = 6; male: *N* = 6) were tested on rewarded alternation in a similar manner as Experiment 1. Rewarded alternation testing started with a preliminary training stage in the absence of any injections. Mice received five trials per day for 6 days. Mice were then tested under the effects of haloperidol or saline in a within‐subjects design. In the first drug‐testing run, half of the mice from each genotype were treated with haloperidol and the remaining mice with saline. Mice received 14 trials of rewarded alternation after injection. After this stage, re‐testing was carried out in the absence of haloperidol or saline, with mice receiving five trials per day for 2 days. In the second drug‐testing run, mice that were previously injected with saline were now treated with haloperidol and vice‐versa, and again they received 14 trials of rewarded alternation after injection. The order of haloperidol/saline administration was counterbalanced across sex as well as genotype. For the drug‐testing runs, testing commenced 15 min after injection of haloperidol/saline. In contrast with Experiment 1, the mice were allowed a maximum of 180 s to complete each of the choice and sample runs. If this time limit was not met, the trial was ended and considered void. Mice that failed to complete at least 10 of the 14 trials when tested under the effects of haloperidol were excluded from all analyses of rewarded alternation performance. Consequently, analysis of the drug testing stages was based on data from the first 10 successfully completed trials per session. The experimenter was blind to the genotype and drug allocations of the animals throughout testing.

### Experiment 4: The effect of haloperidol on spontaneous locomotor activity in Gria1^*−/−*^ mice

Finally, the same mice as used previously in Experiment 3 were tested for spontaneous locomotor activity with haloperidol or vehicle (see Table 1). Although spontaneous locomotor activity was measured in a similar way to Experiment 2, the apparatus used was different. Specifically, mice were placed individually into novel transparent plastic cages (26 × 16 × 17 cm) that were positioned between two sensor panels, with two horizontal photocell beams projecting perpendicularly across the long axis of each cage. The number of beam breaks that each mouse made was recorded by a computer in eight time bins of 15 min each. The session lasted for 2 h. Locomotor testing commenced after the completion of Experiment 3. Mice were put back on a free‐feeding regime 2 weeks before locomotor testing began. Half of the *Gria1* ^*−/−*^ mice and half of the WT mice were injected with haloperidol, and the remaining mice were injected with saline, before they were then immediately placed into the activity cages for 2 h.

### Statistical analyses

Data were analysed using multifactorial anova, or *t*‐tests for between group comparisons. Interactions were analysed using simple main effects analyses using the error term from the original anova. Data were assessed for normality using the Shapiro‐Wilk test. Analysis was conducted using spss software, version 20.

## Results

### Experiment 1A: The group II mGluR agonist LY354740 (15 mg/kg) had no effect on spatial working memory performance in Gria1^*−/−*^ or WT mice

The performance of mice during the pre‐drug training phase was analysed using a 2 (genotype) by 2 (sex) anova. As expected, *Gria1* ^*−/−*^ mice exhibited a clear spatial working memory impairment during the initial pre‐drug testing stage of the rewarded alternation T‐maze task (mean alternation: WT = 70.91% ± 3.24 SEM; *Gria1* ^*−/−*^ mice = 53.11% ± 1.80 SEM; *F*_1,22_ = 24.017 *P* < 0.001). There was no main effect of sex (*F *< 1, *P* > 0.4) or genotype by sex interaction (*F* < 1, *P* > 0.6).

**Figure 1:**
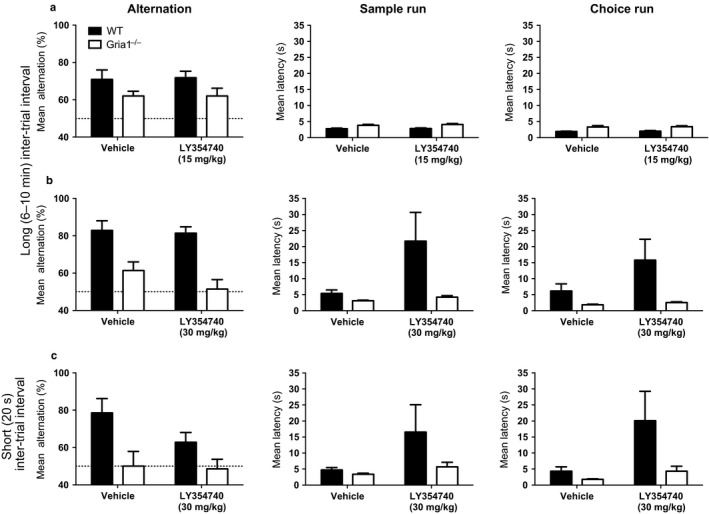
Rewarded alternation performance in WT and *Gria1* ^*−/−*^ mice treated with vehicle and LY354740 in Experiments 1A (panel a; LY354740 15 mg/kg: WT, *N* = 11, Gria1^*−/−*^, *N* = 15), 1B (panel b; LY354740 30 mg/kg: WT, *N* = 7, Gria1^*−/−*^, *N* = 7) and 1C (panel c; LY354740 30 mg/kg: WT, *N* = 7, Gria1^*−/−*^, *N* = 7). In Experiment 1A (panel a) mice were tested using an inter‐trial interval of 6‐10 min. This procedure was repeated in Experiment 1B (panel b), but with a higher dose (30 mg/kg). In Experiment 1C (panel c) the same mice that were tested in Experiment 1B were tested again with the 30 mg/kg dose but the inter‐trial interval was 20 s. The left column shows the mean alternation. The dashed line indicates chance level performance. The middle and right columns show the latencies for the sample run and choice run, respectively. Error bars indicate SEM.

The performance of mice during the drug‐testing phase is shown in Fig. 1a. A 15 mg/kg dose of LY354740 failed to rescue spatial working memory performance in the knockout mice. During the drug‐testing phase one male *Gria1* ^*−/−*^ mouse failed to complete any runs when treated with LY354740, and therefore the data from this mouse were excluded from further analyses. The performance of mice was analysed using a 2 (genotype) by 2 (sex) by 2 (drug/saline) anova. *Gria1* ^*−/−*^ mice were still impaired compared to WT mice (*F*_1,21_ = 4.74, *P* = 0.041) during the drug testing sessions. There was no significant effect of drug and drug did not significantly interact with genotype (both *F* values < 1, *P* > 0.9, see Fig. 1a). There was also no significant main effect of sex (*F* < 1, *P* > 0.60) and sex did not interact with other factors (*F* values < 1, *P* values > 0.30). The 15 mg/kg dose of the drug also had no effect on the running latencies of the mice on either the sample (*F* < 1, *P* > 0.30) or choice runs (*F* < 1 *P* > 0.70), and there was no significant interaction between drug and genotype (*F* < 1, *P* > 0.50). However, *Gria1* ^*−/−*^ mice took longer to complete both sample and choice runs in this particular experiment (*F* values > 10.00, *P* values < 0.005, see Fig. 1a). There were no other significant main effects or interactions (*P* values > 0.10).

### Experiment 1B: The group II mGluR agonist LY354740 (30 mg/kg) had no effect on spatial working memory performance in Gria1^*−/−*^ and WT mice

We next investigated whether a higher dose of the group II mGluR agonist (30 mg/kg) might rescue spatial working memory performance. A separate cohort of experimentally naïve mice were trained on the T‐maze task as before. The results were analysed using a similar analyses as Experiment 1A except the factor of sex was omitted due to all mice being male. The *Gria1* ^*−/−*^ mice were again substantially impaired during the pre‐drug testing phase (mean alternation: WT = 80.95% ± 2.15 SEM; *Gria1* ^*−/−*^ mice = 46.67% ± 2.30 SEM; *F*_1,12_ = 118.72, *P* < 0.001).

The performance of mice during the drug‐testing phase is shown in Fig. 1b. The higher 30 mg/kg dose of LY354740 also failed to rescue rewarded alternation performance in the knockout mice (main effect of drug – *F*_1,12_ = 2.043, *P* = 0.178), and drug treatment did not interact with genotype (*F*_1,12_ = 1.149, *P* = 0.305, see Fig. 1b). The pronounced deficit in the *Gria1* ^*−/−*^ mice remained across the drug test sessions (main effect of genotype, *F*_1,12_ = 24.608, *P* < 0.001). There was a strong suggestion that the 30 mg/kg dose prolonged running latencies in both the sample (*F*_1,12_ = 4.42, *P* = 0.057) and choice runs (*F*_1,12_ = 5.07,  = 0.044). On this occasion the *Gria1* ^*−/−*^ mice ran faster than WT mice, but the effect of genotype failed to reach significance (*F*_1,12_ values < 4.20, *P* values > 0.06). The interaction between drug and genotype also failed to reach significance (*F*_1,12_ values < 3.80, *P* values > 0.07).

### Experiment 1C: The group II mGluR agonist LY354740 (30 mg/kg) had no effect on rewarded alternation testing with a short inter‐trial interval in Gria1^*−/−*^ and WT mice

Given the lack of effect of the group II mGluR agonist on spatial working memory performance in Experiments 1A and 1B, the T‐maze testing paradigm was changed to more closely resemble that used by Moghaddam & Adams (1998), in which LY354740 had been shown previously to ameliorate the effect of the non‐competitive NMDAR antagonist, PCP, on rewarded alternation. This was achieved by reducing the inter‐trial interval from 6‐10 min to 20 s. The pronounced spatial memory deficit in the *Gria1* ^*−/−*^ mice was still evident with this short inter‐trial interval during initial ‘pre‐drug’ testing (WT = 73.33% ± 4.24 SEM; *Gria1* ^*−/−*^ mice = 49.52% ± 3.89 SEM; *F*_1,12_ = 17.16, *P* = 0.001). However, LY354740 still failed to rescue memory performance when using this modified paradigm during the drug test sessions. There was still no significant main effect of drug (*F*_1,12_ = 1.28, *P* = 0.28), and no interaction between drug and genotype (*F* < 1, *P* > 0.30). There was an overall main effect of genotype (*F*_1,12_ = 15.52, *P* = 0.002, see Fig. 1c), reflecting the continued and persistent short‐term/working memory deficit in the knockout mice.

The 30 mg/kg dose of LY354740 did, nevertheless, result in significantly prolonged running latencies on the choice run (*F*_1,12_ = 4.83, *P* = 0.048, see Fig. 1c). There was a similar pattern of results for the sample run latencies but this failed to reach significance (*F*_1,12_ = 3.034, *P* = 0.107). The effect of drug did not significantly interact with genotype (*F*_1,12_ values < 2.60, *P* values > 0.10), and there was no significant main effect of genotype for running latencies in this experiment (*F*_1,12_ values < 3.20, *P* values ≥ *P* 0.10).

### Experiment 2: The group II mGluR agonist LY354740 reduced spontaneous locomotor activity in wild‐type and Gria1^*−/−*^ mice

We next determined whether LY354740 would affect the locomotor hyperactivity in a novel environment in *Gria1* ^*−/−*^ mice. The mGluR agonist reduced activity levels in both wild‐type and *Gria1* ^*−/−*^ mice.

**Figure 2:**
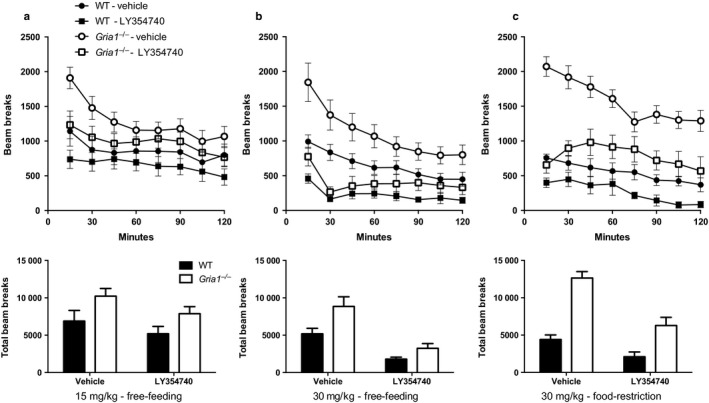
Spontaneous locomotor activity in WT and *Gria1* ^*−/−*^ mice treated with vehicle and LY354740 in Experiments 2A (panel a; LY354740 15 mg/kg: WT, *N* = 11, Gria1^*−/−*^, *N* = 15), 2B (panel b; LY354740 30 mg/kg: WT, *N* = 12, Gria1^*−/−*^, *N* = 11) and in 2C (panel c; LY354740 30 mg/kg: *N* = 7 per group). In Experiment 2A mice were tested while under a free‐feeding regime. The procedure was repeated in Experiment 2B, but with a higher dose (30 mg/kg). In Experiment 2C mice that were tested in Experiment 1B and 1C were tested with the 30 mg/kg dose, but in contrast to Experiment 2B, mice were now tested under food restriction (85% of free‐feeding weights), thus matching the conditions under which rewarded alternation performance was tested. The top row shows the number of beam breaks in a 2‐h period in bins of 15 min. Error bars indicate ± SEM. The bottom row shows the total number of beam breaks in the 2 h session. Error bars indicate SEM.

In Experiment 2A spontaneous locomotor activity was analysed using a 2 (genotype) by 2 (sex) by 2 (drug) by 8 (time bin) anova. It was found that *Gria1* ^*−/−*^ mice were hyperactive compared to WT mice (*F*_1,22_ = 5.82, *P* = 0.025, see Fig. 2a). The 15 mg/kg dose of LY354740 reduced locomotor activity (*F*_1,22_ = 5.24, *P* = 0.032), but the effect of drug did not interact with genotype (*F* < 1, *P* > 0.6). Activity declined over time bins (*F*_7,154_ = 15.74, *P* < 0.001) and the effect of genotype significantly interacted with time bin (*F*_7,154_ = 2.32, *P* = 0.028), as did drug (*F*_7,154_ = 2.76, *P* = 0.01) and sex (*F*_7,154_ = 2.74,  = 0.01), with the decline in activity over time being greater for male than female mice. There were no other significant main effects or interactions of factors (*P* values > 0.1).

The results of Experiment 2B were analysed in the same manner as for Experiment 2A. In Experiment 2B *Gria1* ^*−/−*^ mice were again hyperactive (*F*_1,19_ = 9.13, *P* = 0.007) and, not surprisingly, the 30 mg/kg dose of LY354740 substantially reduced activity (*F*_1,19_ = 50.54, *P* < 0.001), but, as before, the effect of drug did not significantly interact with genotype (*F*_1,19_ = 2.70, *P* = 0.18, see Fig. 2b). There was a three‐way genotype, sex and drug interaction (*F*_1,19_ = 5.45, *P* = 0.031). This interaction, however, reflected a failure to find the usual significant hyperactivity effect in male *Gria1* ^*−/−*^ mice in the vehicle condition in this particular experiment. Once again the effects of genotype, sex and drug each interacted with time bin (*F* values > 2.80, *P* values < 0.05).

It is possible that the effect of LY354740 in successfully reducing the locomotor hyperactivity in *Gria1* ^*−/−*^ mice, but the lack of any drug effect on spatial working memory performance, could reflect the different motivational/hunger state of the mice in the two experiments (food deprived for spatial working memory testing vs. *ad libitum* food for locomotor testing). To investigate this possibility, we assessed the effects of LY354740 on spontaneous locomotor activity levels in wild‐type and *Gria1* ^*−/−*^ mice maintained under the same food restriction schedule as used for spatial working memory testing.

The results of Experiment 2C were analysed in a similar manner to Experiments 2A and 2B except that effect of sex was omitted due to all mice being male. A 30 mg/kg dose of LY354740 again significantly reduced locomotor activity (*F*_1,12_ = 51.26, *P* < 0.001). *Gria1* deletion resulted in pronounced hyperactivity (*F*_1,12_ = 38.80, *P* < 0.001), and on this occasion the effect of drug significantly interacted with genotype (*F*_1,12_ = 11.30, *P* = 0.006, see Fig. 2c). The interaction reflected the fact that the effect of drug was larger for *Gria1* ^*−/−*^ mice (*F*_1,12_ = 55.35, *P* < 0.001) than for WT mice (*F*_1,12_ = 7.21, *P* = 0.02). There was also a significant effect of time bin (*F*_7,84_ = 15.55, *P* < 0.001) and significant three‐way interaction of factors (*F*_7,84_ = 3.60, *P* = 0.002).

### Experiment 3: Haloperidol had no effect on rewarded alternation in Gria1^*−/−*^ or wild‐type mice

For comparison with the LY354740 data, we also investigated the effects of the anti‐psychotic D2 receptor antagonist, haloperidol, on spatial working memory performance in *Gria1* ^*−/−*^ mice. Previously, Wiedholz *et al*. (2008) have demonstrated that haloperidol (0.3 mg/kg) reduces the locomotor hyperactivity exhibited by *Gria1* ^*−/−*^ mice. However, it is also important to note that haloperidol also decreased activity levels in normal animals (Wiedholz *et al*., 2008). In addition, *in vivo* chronoamperometric measurements showed retarded dopamine clearance in the knockouts, consistent with the possibility of a hyperdopaminergic state. More recently, we have used fast‐scan cyclic voltammetry to directly demonstrate increased phasic dopamine transients in freely moving, behaving *Gria1* ^*−/−*^ mice relative to wild‐type littermates (Boerner *et al*., in preparation). Therefore, there is evidence for a hyper‐dopaminergic phenotype in these animals, and the D2 antagonist attenuates the locomotor hyperactivity in the knockouts. We therefore investigated whether haloperidol would have any effect on spatial working memory performance in *Gria1* ^*−/−*^ mice.

Five WT and three *Gria1* ^*−/−*^ mice failed to complete at least 10 out of 14 trials of rewarded alternation when tested under the effects of haloperidol and were thus removed from all analyses of rewarded alternation, including the initial, drug‐free testing stage (leaving WT, *N* = 7 and *Gria1* ^*−/−*^, *N* = 9). A two‐tailed chi‐square test demonstrated that the proportions of mice excluded for each genotype did not differ, χ²(1) = 0.75, *P* = 0.39.

The results of the initial training prior to drug treatment were analysed using a 2 (genotype) by 2 (sex) anova. It was found that *Gria1* ^*−/−*^ mice were again significantly impaired on the rewarded alternation task (mean alternation = 52.59% ± 3.71 SEM) compared to WT mice (mean alternation = 69.52% ± 4.79 SEM; *F*_1,12_ = 10.31, *P* = 0.007). There was no significant main effect of sex on choice performance (*F* < 1, *P* > 0.3). Due to the lack of effect of sex during the initial testing state the factor of sex was removed from subsequent analyses.

**Figure 3:**
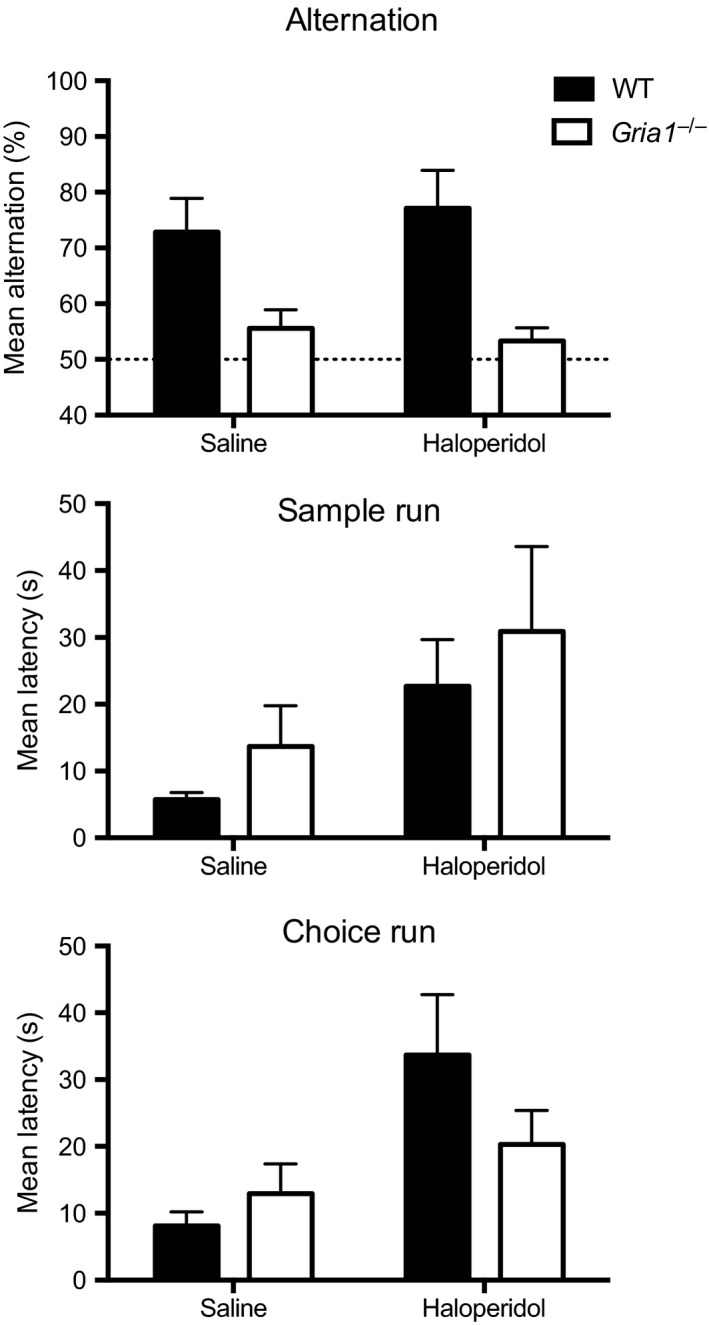
Rewarded alternation performance in WT (*N* = 7) and *Gria1* ^*−/−*^ (*N* = 9) mice treated with saline and haloperidol (0.3 mg/kg) in Experiment 3. The top panel shows the mean alternation. The dashed line indicates chance level performance. The middle and bottom panels show the latencies for the sample run and choice run, respectively. Error bars indicate SEM.

Haloperidol treatment did not result in a significant change in the choice accuracy (percent correct choices) of either WT or *Gria1* ^*−/−*^ mice (main effect of drug: *F* < 1, *P* > 0.7, see Fig. 3). As during the initial drug‐free testing, the effect of genotype on choice performance was highly significant, reflecting the pronounced short‐term/working memory impairment in the *Gria1* ^*−/−*^ mice (*F*_1,14_ = 13.52, *P *=* *0.002). There was no significant drug by genotype interaction (*F* < 1, *P* > 0.3).

Haloperidol treatment did have a significant effect on time taken to traverse the maze. There was a significant increase in both sample run (effect of drug, *F*_1,14_ = 10.99, *P* = 0.005) and choice run latencies (*F*_1,14_ = 19.78, *P *=* *0.001, see Fig. 3). There was no significant effect of genotype on either sample (*F* < 1, *P *>* *0.4) or choice latencies (*F* < 1, *P *>* *0.5). However, there was a significant drug by genotype interaction for choice latencies (*F*_1,14_ = 6.04, *P *=* *0.028). Simple main effects analysis revealed that haloperidol significantly prolonged choice latencies for the WT mice (*F*_1,14_ = 21.19, *P *<* *0.001), but not for *Gria1* ^*−/−*^ mice (*F*_1,14_ = 2.27, *P *=* *0.16). The drug by genotype interaction for sample latencies was not significant (*F* < 1, *P* > 0.9).

### Experiment 4: Haloperidol reduced spontaneous locomotor activity in wild‐type and Gria1^*−/−*^ mice

**Figure 4:**
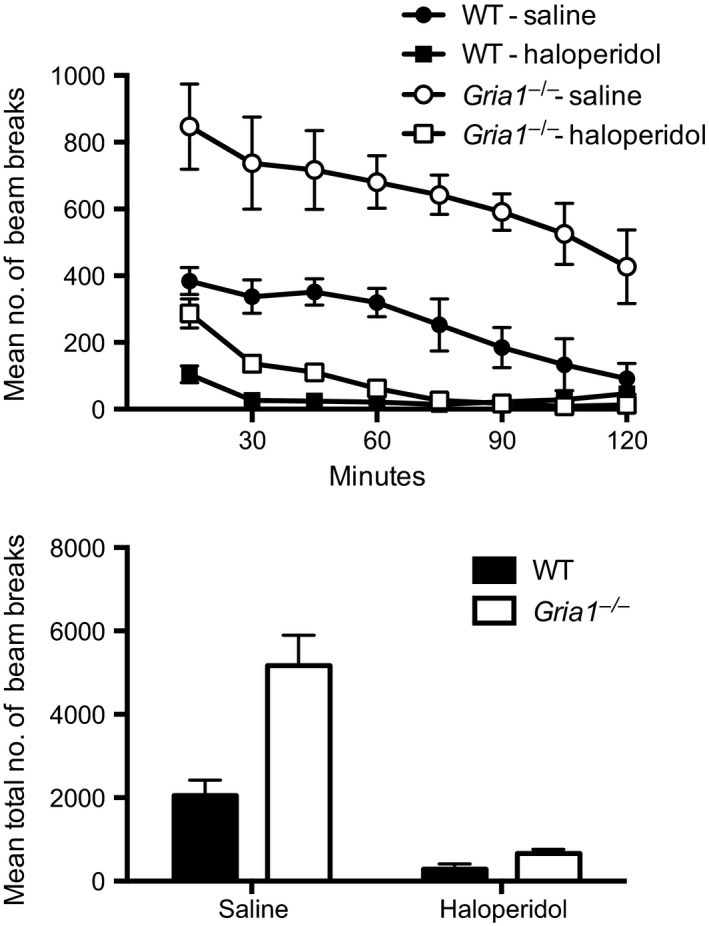
Spontaneous locomotor activity in WT and *Gria1* ^*−/−*^ mice treated with either haloperidol (0.3 mg/kg) or saline (*N* = 6 per drug per genotype) in Experiment 4. The top panel shows the number of beam breaks in a 2‐h period in bins of 15 min. Error bars indicate ± SEM. The bottom panel shows the total number of beam breaks for the 2‐h period of testing. Error bars indicate SEM.

Finally, we revisited the effects of the D2 receptor antagonist on the locomotor hyperactivity in *Gria1* ^*−/−*^ mice. The results were analysed in a similar manner to Experiment 2 except that the factor of sex was omitted due to the numbers of each sex within each genotype and drug allocation being small (*N* = 3). Haloperidol treatment significantly reduced spontaneous locomotor activity (main effect of drug, *F*_1,20_ = 56.20, *P *<* *0.001, see Fig. 4). There was also a significant main effect of genotype (*F*_1,20_ = 17.37, *P *<* *0.001), and a significant genotype by time bin interaction (*F*_7,140_ = 2.79, *P *<* *0.05), reflecting the pronounced hyperactivity in the *Gria1* ^*−/−*^ mice. There was also a drug by time bin interaction (*F*_1,140_ = 7.38, *P* < 0.001) and a significant genotype by drug interaction (*F*_1,20_ = 10.74, *P *=* *0.004). Simple main effects analysis showed that while saline treated *Gria1* ^*−/−*^ mice displayed significant novelty‐induced hyperlocomotion compared to saline treated WT mice (*F*_1,20_ = 27.72, *P *<* *0.001), there was no significant difference in novelty‐induced locomotor activity between haloperidol treated *Gria1* ^*−/−*^ and WT mice (*F* < 1, *P *>* *0.5). Furthermore, simple main effects analysis also showed that haloperidol significantly reduced activity levels in both WT (*F*_1,20_ = 8.90, *P* = 0.007) and *Gria1* ^*−/−*^ mice (*F*_1,20_ = 58.05, *P* < 0.001). There was no significant three‐way interaction of factors (*F*_7,150_ < 1, *P* = 0.55).

### Acquisition of rewarded alternation performance in Experiments 1a, 1b and 3

An additional analysis of acquisition of rewarded alternation performance, pooled across experiments 1a, 1b 3, in which mice were experimentally naïve at the start of training, showed that WT mice started substantially above chance (first 10 trials, mean = 72.00% correct ± 2.15 SEM, *t*_24_ = 6.84, *P* < 0.001, chance = 50%). This is consistent with other demonstrations that mice spontaneously alternate rather than actually acquiring the alternation behaviour over training (see Sanderson & Bannerman, 2012, for a discussion of how alternation performance reflects short‐term habituation rather than acquisition of an alternation rule). In contrast, *Gria1* ^*−/−*^ mice failed to perform above chance (first 10 trials, mean = 50.32% correct ± 3.21 SEM, *t*_30_ < 1, *P* > 0.80). Furthermore, both genotypes did not significantly improve over subsequent blocks of trials (second block of 10 trials: WT mean = 73.60% correct ± 2.51 SEM, *Gria1* ^*−/−*^ mean = 50.32% correct ± 2.64 SEM; third block of 10 trials: WT mean = 74.40% correct ± 3.32 SEM, *Gria1* ^*−/−*^ mean = 53.87% correct ± 2.35 SEM; effect of block: *F* < 1, *P* > 0.40; block by genotype interaction: *F* < 1, *P* > 0.80; effect of genotype (*F*_2,54_ = 71.27, *P* < 0.001).

## Discussion

The group II mGluR agonist, LY354740, reduced locomotor activity levels in both wild‐type and *Gria1* ^*−/−*^ mice but, notably, it did not rescue spatial short‐term/working memory performance in the knockout animals, regardless of sex, during testing on the T‐maze rewarded alternation task. Similarly, we found that the dopamine D2 receptor antagonist haloperidol reduced the locomotor hyperactivity observed in *Gria1* ^*−/−*^ mice when they were placed in a novel environment, but the drug had no effect on choice accuracy performance levels during spatial short‐term/working memory testing in either knockout mice or controls, despite again producing clear motoric effects on the T‐maze.

### Group II mGluR agonist LY354740 fails to rescue short‐term memory performance in a genetic mouse model of glutamatergic hypofunction

The group II mGluR agonist LY354740 had no effect on spatial working/short‐term memory performance during T‐maze rewarded alternation testing in *Gria1* ^*−/−*^ mice, indicating an inability to rescue cognitive performance in a schizophrenia‐relevant genetically modified mouse model of glutamatergic dysfunction. Neither 15 nor 30 mg/kg doses of the drug had any effect on choice accuracy, despite both doses resulting in clear reductions in locomotor activity levels in both genotypes. Therefore, these drug doses were not without effect. There were clear reductions in locomotor activity that were not accompanied by any rescue of spatial short‐term memory processes. The lack of any rescue effect in the *Gria1* ^*−/−*^ mice is unlikely to be due to any negative performance effects of LY354740 masking any positive effects of the drug on memory performance, as the drug was also without any significant effect in the wild‐type controls.

The failure of a group II mGluR agonist to rescue the spatial working memory deficit in a genetic model of glutamatergic hypofunction (*Gria1* ^*−/−*^ mice), stands in contrast with the successful rescue of the deficit with pharmacological models of glutamatergic hypofunction, for example with non‐competitive NMDAR antagonists such as phencyclidine or MK801 (Moghaddam & Adams, 1998; Blot *et al*., 2015). There are several overt differences between these models (genetic vs. pharmacological; AMPARs vs. NMDARs; constitutive knockout throughout the lifetime of the animal vs. acute drug administration; receptor subunit ablation vs. use‐dependent channel blockade), many, or all, of which could contribute to the different outcomes with mGluR agonists. Further studies, such as assessment of the effects of LY354740 on spatial working memory deficits in genetic models of NMDAR hypofunction (e.g., Niewoehner *et al*., 2007; Bannerman *et al*., 2008; Korotkova *et al*., 2010), would shed further light on this issue.

Indeed, it would seem important to assess further the generality of the effects of group II mGluR agonists on spatial working memory performance in other models of glutamatergic dysfunction and, specifically, NMDAR hypofunction. It is potentially of note that both of the published reports involve non‐competitive, NMDAR channel blockers (PCP or MK‐801). These drugs exhibit a use‐dependent blockade of the NMDAR channel which reflects the fact that a certain amount of glutamatergic activity is required in order to open the NMDAR channels and allow the blockers access to their binding sites. It is possible that the group II mGluR agonists, which are known to reduce glutamate release through their action on presynaptic autoreceptors, could indirectly result in less NMDAR channel openings and thus less opportunity for the NMDAR antagonists to access their binding sites. This could reduce the effectiveness of these non‐competitive NMDAR antagonists in the presence of drugs like LY354740. Interestingly, one might make the opposite prediction for competitive antagonists like AP5 or CPP, where any reduction in pre‐synaptic glutamate release caused by a group II mGluR agonist might actually make the competitive antagonists more efficacious as they will have less glutamate to compete with for access to receptor binding sites. Either way, it would be desirable to determine whether group II mGluR agonists like LY354740 can rescue spatial working memory deficits with other NMDAR manipulations, including both genetic models (Nakazawa *et al*., 2003; Niewoehner *et al*., 2007; Korotkova *et al*., 2010) and other pharmacological manipulations of NMDAR function such as competitive antagonists (McHugh *et al*., 2008). This could be of particular relevance for judging the clinical applicability of previous findings.

### Haloperidol does not affect spatial short‐term/working memory

The spatial short‐term/working memory performance of both wild‐type and *Gria1* ^*−/−*^ mice, in terms of choice accuracy on the T‐maze rewarded alternation task, was also unaffected by haloperidol treatment, even despite the clear motoric effects of the drug seen during testing. The absence of a haloperidol effect on T‐maze choice accuracy in normal animals has been shown previously (Aultman & Moghaddam, 2001), and was not because the dose of the drug was ineffective in the present study. The same dose of haloperidol had a marked effect on locomotor activity levels in both genotypes in the photocell activity cages, which replicated the previous study by Wiedholz *et al*. (2008). Furthermore, the drug also had clear motoric effects during the T‐maze task itself. Haloperidol prolonged the running latencies of the mice (on both the sample and choice runs), despite having no effect on memory performance. This led to some animals failing to complete the trials in the allotted time and ‘timing out’, although it is important to note that this effect was equivalent for both genotypes. We cannot rule out the possibility that a higher dose of haloperidol may have an effect on spatial short‐term/working memory but it is also worth noting that any further increase in the dose of the dopamine receptor antagonist used is likely to have resulted in the animals failing to run and perform the task. Indeed, as mentioned above, even at the dose of 0.3 mg/kg used in the present study, five wild‐type and three *Gria1* ^*−/−*^ mice failed to complete the experiment and had to be excluded from the study. There is the distinct possibility that at higher doses the experiment would prove unworkable. Nevertheless, the present study clearly shows that haloperidol has motoric effects in both wild‐type and *Gria1* ^*−/−*^ mice, without having any effect on spatial short‐term/working memory.

In theory, it is also conceivable that a lower dose of haloperidol could have a beneficial effect on spatial short‐term memory performance in *Gria1* ^*−/−*^ mice, and that the 0.3 mg/kg dose of haloperidol that we used was too high, resulting in non‐mnemonic effects that were detrimental to performance, thus masking any positive effects that the drug might have had on memory in the knockouts. In other words, it is possible that haloperidol could rescue short‐term memory function in GluA1 knockout mice but the motoric effects of the drug prevent that rescue from being expressed at the behavioural level in these animals. However, we think that this possibility is extremely unlikely given that the same dose of haloperidol also had absolutely no effect on T‐maze choice accuracy in the wild‐type controls. There is therefore no evidence for any detrimental effects of haloperidol in terms of choice accuracy memory performance on the T‐maze task.

The fact that haloperidol failed to affect spatial short‐term/working memory in these experiments is in contrast to reports that dopamine receptor blockade can impair reference memory tasks assessing associative, long‐term spatial memory (Whishaw & Dunnett, 1985; Ploeger *et al*., 1992; Gasbarri *et al*., 1996; Mura & Feldon, 2003). We have argued elsewhere that performance on the win‐shift, rewarded alternation, T‐maze task likely relies on a non‐associative, short‐term memory trace which provides a sense of relative familiarity based on recent experience (Sanderson & Bannerman, 2012). The present experiments would appear to suggest that this form of non‐associative short‐term spatial memory is not dopamine‐dependent.

## Conclusions

In summary, the group II mGluR agonist LY354740 and the dopamine D2 receptor antagonist haloperidol reduced the locomotor hyperactivity seen in *Gria1* ^*−/−*^ mice but had no effect on the spatial short‐term/working memory deficit in these animals. These data suggest that both LY354740 and haloperidol may act by reducing the expression of behaviour in general, thus silencing any behavioural phenotype without correcting any underlying deficit in cognition. This is reminiscent of the clinical situation, in which existing antipsychotic medications are effective against psychotic symptoms but have little impact on the cognitive impairments, which are widely considered to lie at the heart of the disorder (Kahn & Keefe, 2013; Fatouros‐Bergman *et al*., 2014).

## Conflict of interest

Gary Gilmour is an employee of Eli Lilly & Co. Ltd.

## Author contributions

The experiments were designed by T.B., A.F., D.M.B. and D.J.S. The data were collected and analysed by T.B., A.B., J.C., A.F., S.J., C.B. and D.J.S. The paper was written by T.B., R.S., P.H.S., P.J.H., G.G., D.M.B. and D.J.S.

## Data accessibility

On acceptance for publication the raw data will be deposited on the website figshare.com.
